# A Novel Isoprene Synthase from the Monocot Tree *Copernicia prunifera* (Arecaceae) Confers Enhanced Drought Tolerance in Transgenic Arabidopsis

**DOI:** 10.3390/ijms242015329

**Published:** 2023-10-18

**Authors:** Jiamei Yu, Iuliia Khomenko, Franco Biasioli, Mingai Li, Claudio Varotto

**Affiliations:** 1 Biodiversity, Ecology and Environment Area, Research and Innovation Centre, Fondazione Edmund Mach, San Michele all’Adige, 38098 Trento, Italy; jiamei.yu19@outlook.com; 2 Department of Biology, University of Padova, 35121 Padova, Italy; 3 Food and Nutrition Area, Research and Innovation Centre, Fondazione Edmund Mach, San Michele all’Adige, 38098 Trento, Italy; iuliia.khomenko@fmach.it (I.K.); franco.biasioli@fmach.it (F.B.); 4 National Biodiversity Future Center (NBFC), 90133 Palermo, Italy

**Keywords:** isoprene synthase, drought stress, Arecaceae, isoprene emission, drought tolerance

## Abstract

The capacity to emit isoprene, among other stresses, protects plants from drought, but the molecular mechanisms underlying this trait are only partly understood. The Arecaceae (palms) constitute a very interesting model system to test the involvement of isoprene in enhancing drought tolerance, as their high isoprene emissions may have contributed to make them hyperdominant in neotropical dry forests, characterized by recurrent and extended periods of drought stress. In this study we isolated and functionally characterized a novel *isoprene synthase*, the gene responsible for isoprene biosynthesis, from *Copernicia prunifera*, a palm from seasonally dry tropical forests. When overexpressed in the non-emitter *Arabidopsis thaliana*, *CprISPS* conferred significant levels of isoprene emission, together with enhanced tolerance to water limitation throughout plant growth and development, from germination to maturity. *CprISPS* overexpressors displayed higher germination, cotyledon/leaf greening, water usage efficiency, and survival than WT Arabidopsis under various types of water limitation. This increased drought tolerance was accompanied by a marked transcriptional up-regulation of both ABA-dependent and ABA-independent key drought response genes. Taken together, these results demonstrate the capacity of *CprISPS* to enhance drought tolerance in Arabidopsis and suggest that isoprene emission could have evolved in Arecaceae as an adaptive mechanism against drought.

## 1. Introduction

Isoprene (2-methyl-1,3-butadiene, C_5_H_8_) is the single most abundant biological volatile organic compound (BVOC) with an estimated annual emission of around 500 Tg of carbon (C) year^−1^, approximately equaling that of methane and making it the main hydrocarbon released from plants into the atmosphere [1,2]. In addition to plants, the largest natural sources of isoprene emissions, other organisms such as fungi, bacteria, and animals also emit isoprene [3], but trees from tropical areas are widely recognized as the major contributors to biological isoprene emission [4]. In plants, isoprene biosynthesis is catalyzed by the enzyme isoprene synthase (ISPS) using as substrate the dimethylallyl diphosphate (DMADP) generated through the methylerythritol 4-phosphate (MEP) pathway in chloroplasts and requiring either Mg^2+^ or Mn^2+^ as main cofactors [5,6]. Thus, at the protein level, isoprene biosynthesis is ultimately regulated by the activity of ISPS and the availability of DMADP in both dicot and monocot natural emitters [7,8,9,10,11,12]. In turn, as DMADP in chloroplasts is mainly generated through photosynthesis and the enzyme shows a strong temperature-dependent activity, light and temperature are among the most significant environmental factors to influence isoprene emission by ISPS [13]. Transcriptional control of the *ISPS* gene has also been shown to provide a further level of regulation for isoprene emission in response to stress [7,14,15,16,17,18,19]. Finally, *ISPS* transcription is the most likely factor determining the developmental regulation of isoprene emission in poplar leaves [7,20], in agreement with physiological data in both dicot and monocot natural emitters.

The multiple and partly redundant levels of ISPS activity regulation are motivated by the fact that the emission of isoprene is a metabolically expensive process requiring significant amounts of energy and reducing power [13]. Moreover, significant fractions of photosynthetically fixed carbon (C) can be released as isoprene (e.g., [21,22,23]). Therefore, continuous isoprene emission under stress conditions causes a net C and energy loss. However, a relatively high number of plant species (estimated as around 20% of angiosperm scattered in different families) preferentially emit isoprene [24], suggesting that this trait can provide an evolutionary advantage for particular species and/or for environmental conditions [25,26].

Indeed, the beneficial effects of isoprene emission against both biotic and abiotic stresses have been repeatedly confirmed by several studies. Isoprene has been reported to protect plants against damages caused by herbivores and pathogens [27,28], although it can also deter some herbivore parasitic wasps [29]. Based on this dual role of isoprene in stress tolerance, it has been recently proposed that in evolutionary terms isoprene emission may mediate the growth–defense tradeoff in the face of climate stress [30]. Concerning abiotic stresses, the enhanced tolerance to high temperature and the oxidative stress caused by ozone and other reactive oxygen species (ROS) are considered among the main functions of isoprene emission from plants [1,24,31,32,33,34,35,36]. Furthermore, several lines of evidence suggest that isoprene emitting genotypes or species can better withstand drought stress than their closely related non-isoprene emitting counterparts [12,37]. Even in heterologous systems, the engineering of isoprene emission resulted in enhanced water use efficiency and protection of the photosynthetic apparatus, although at the cost of reduced growth [38]. As the two unsaturated carbon–carbon bonds of the isoprene molecule make it highly reactive towards ROS [39], isoprene has been suggested to directly act as an antioxidant and protect cellular components from the oxidative stress and the consequent damage resulting from drought stress [38,40]. In various plant systems, isoprene (either exogenously supplied or endogenously produced) has been shown to induce a complex series of transcriptomic, proteomic, and metabolomic modifications. Some of these changes seem to improve drought tolerance, in particular through a higher photosynthetic performance and remodeling of protein abundance in the chloroplast, as well as an altered membrane trafficking accompanied by reduced oxidative stress and increased concentrations of abscisic acid (ABA) and osmoprotectants like trehalose and proline [41]. This suggests an isoprene function in signaling and stress priming [42,43,44].

While the majority of the studies mentioned above on the capacity of isoprene emission to mitigate the adverse effects of drought stress has been carried out on either dicotyledonous species or their *ISPS* genes, only a limited number of studies involving monocotyledons has been published until now. Only a handful of isoprene synthase genes were identified and functionally characterized in the monocot families Poaceae (*Arundo donax* L.; *Arundo plinii* Turra; [16,45,46,47]), and recently Arecaceae (*Bismarckia nobilis* Hildebrandt and H. Wendl., *Howea forsteriana* (F. Muell.) Becc., *Phoenix canariensis* H. Wildpret, *Sabal minor* (Jacq.) Pers., *Trachycarpus oreophilus* Gibbons and Spanner, and *Washingtonia filifera* (Rafarin) H. Wendl.; *Phoenix dactylifera* L. [48,49]). Thanks to these studies in monocots, two brand new sets of amino acid tetrads that are diagnostic of ISPS activity were identified, demonstrating for the first time the pivotal role of a single amino acid residue for the evolution of isoprene synthases and further explaining the evolutionary lability of this plant trait with a global relevance [16]. Among the monocot isoprene synthases, *AdoISPS* is able to confer enhanced drought tolerance, accelerated stomatal closure, and a more conservative water usage strategy when overexpressed in *A. thaliana* [45,46]. These results are in line with the in vivo observation that the ISPS enzyme from *Phoenix dactylifera* is strongly upregulated by the combination of heat and drought stresses [49], adding further support to the direct involvement of isoprene in drought tolerance.

In the present study we identified a novel *ISPS* gene from the monocot tree species *Copernicia prunifera* (Miller) H. E. Moore, a palm native to the Caatinga biome, one of the largest areas of the South American seasonally dry tropical forest, where it is widely cultivated to produce carnauba wax. Carnauba is a commercially valuable natural product used in coatings, cosmetics, pharmaceuticals, food products, electronics, and polishing waxes with an estimated induct of USD 55 million per year [50]. *C. prunifera* is highly resistant to the prolonged absence of water alternating with permanent floods [51], and like the majority of Arecaceae species it emits significant amounts of isoprene [48]. With the aim of deepening the current understanding of the function of isoprene emission in drought stress mitigation in this important family of monocotyledonous trees, we characterized the *CprISPS* isoprene synthase gene from *C. prunifera* through its overexpression in the model plant species *Arabidopsis thaliana*.

## 2. Results

### 2.1. CprISPS Is a Novel, Functional Isoprene Synthase Gene from C. prunifera

**Figure 1:**
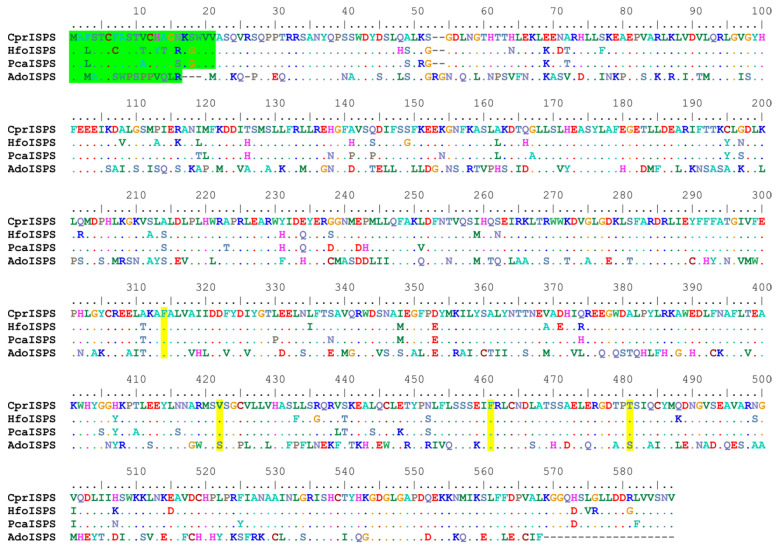
Multiple protein sequence alignment of the novel CprISPS from *C. prunifera* with already identified ISPSs from *Howea forsteriana* (HfoISPS), *Phoenix canariensis* (PcaISPS) and *Arundo donax* (AdoISPS). The predicted chloroplast transit peptides are shown in green, and the amino acids of diagnostic tetrad are marked in yellow. Dashes in the alignment indicate gaps, and dots represent amino acids identical to the first sequence along the alignment. Color-coding of the amino-acids conventionally reflects similarities in their physicochemical properties according to defaults of the Bioedit program (e.g., positively charged AA are coded in dark blue, negatively charged ones are in red, single-ring aromatic ones are in sea-green, etc.).

The newly identified *ISPS* gene from *C. prunifera* (from here on called *CprISPS*) codes for a predicted protein of 585 amino acids, the same length of the previously identified orthologs from other Arecaceae species (Figure 1). The protein shares a sequence identity of 91.79% with PcaISPS from *P. canariensis* (also from subfamily Coryphoideae), of 90.43% with HfoISPS of *H. forsteriana* (subfamily Arecoideae; [48,49]) and of 51.55% identity with AdoISPS from *A. donax*, another monocot species from the Poaceae family that has been characterized in detail [16]. As for the other Arecaceae ISPS, the first 21 amino acids of CprISPS are predicted to act as the transit peptide for chloroplast import based on the program ChloroP 1.1 [52] (Figure 1). CprISPS shares the same diagnostic tetrad of amino acids of all other ISPS from Arecaceae identified so far: the two essential diagnostic residues (F312 and F459) present in all plant isoprene synthases validated until now, the V420 residue shared with some dicotyledon families (Casuarinaceae, Convolvulaceae, Elaeocarpaceae, and Fagaceae) and the Arecaceae-specific residue T479 [16,48].

**Figure 2:**
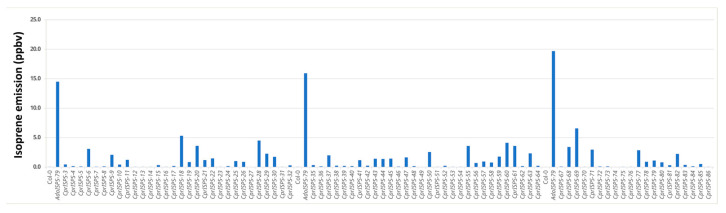
Isoprene emission measurement of 80 transgenic plants overexpressing *CprISPS*. PTR-TOF-MS was used to detect isoprene emission at mass 69.069 from one-month-old plants. *AdoISPS-79* was used as a positive isoprene emitter, and Col-0 as a non-emitter. Ppbv indicates parts per billion volume.

To determine whether the putative *CprISPS* is the isoprene synthase functional orthologue in *C. prunifera*, the full-length cDNA of *CprISPS* was transformed into isoprene non-emitter Arabidopsis Col-0 wild-type plants under the transcriptional control of the CaMV 35S promoter. Integration of the construct for *CprISPS* overexpression in transgenic Arabidopsis plants was first confirmed by PCR. The presence of the typical signature of isoprene emission at molecular mass 69.069 was confirmed with PTR-Tof-MS by assessing 80 independent transgenic lines compared with Col-0 as a reference non-emitter control and the *AdoISPS-79* transgenic line from *A. donax* used in previous studies as an isoprene emitter positive control [16,45]. Statistical analysis indicated that the isoprene emission from these transgenic lines was significantly higher relative to Col-0 wild-type plants (Student’s *t*-test *p* < 0.01) (Figure 2), thus demonstrating that *CprISPS* is a functional isoprene synthase identified from *C. prunifera*. Expression of *CprISPS* was confirmed by semi-quantitative RT-PCR in two transgenic lines with high isoprene emission (*CprISPS-60* and *CprISPS-68*; Supplementary Figure S1), which were then used for all downstream analyses.

### 2.2. Overexpression of CprISPS Enhances Seeds Germination and Cotyledon Greening Rate

**Figure 3:**
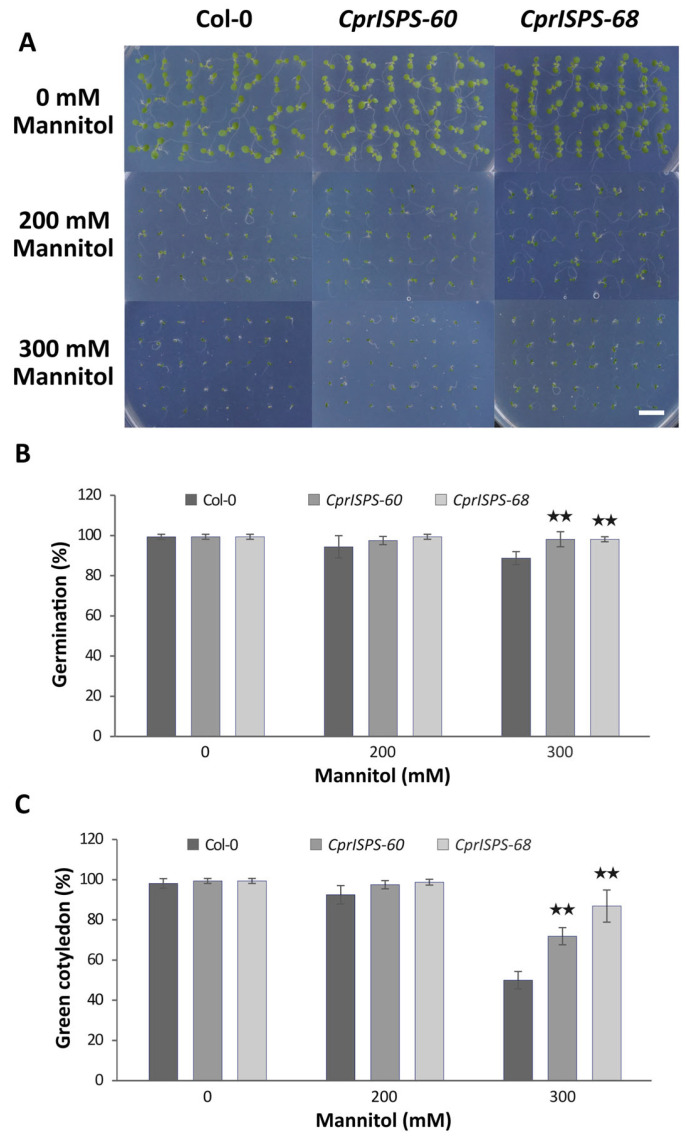
Germination analysis of transgenic lines overexpressing *CprISPS* gene upon mannitol treatment. (**A**) Representative pictures of germinating Col-0 and transgenic plants treated with different concentrations of mannitol (0 mM, 200 mM, 300 mM) for seven days. (**B**) Germination percentage. (**C**) Greening of cotyledons of Col-0 and transgenic plants subjected to different concentrations of mannitol (0 mM, 200 mM, 300 mM) for seven days. Histogram bars marked with two stars (★★) are significantly different from Col-0 (Student’s *t*-test, *p* < 0.01). Error bars represent the SD of the means. Scale bar = 1 cm.

A previous study showed that transgenic Arabidopsis plants overexpressing *AdoISPS* from *A. donax* enhanced the seed germination and cotyledon greening rate when seedlings were subjected to ABA treatment, a major phytohormone involved in diverse responses of abiotic stresses through control of seed dormancy and stomatal closing [46,53]. To investigate the role of *CprISPS* in plant responses to mannitol-induced water stress caused by water-potential reduction in the growth medium, two homozygous single-copy *CprISPS* transgenic lines with high isoprene emission, *CprISPS-60* and *CprISPS-68*, were selected for further studies. Seed germination rates and cotyledon greening rates were examined among *CprISPS-60*, *CprISPS-68,* and Col-0 wild-type plants treated with different concentrations of mannitol (0 mM, 200 mM, and 300 mM). These analyses did not detect any significant differences among samples under control conditions and 200 mM mannitol treatment at day seven after germination, although there was a tendency towards an increased germination rate for both *CprISPS* transgenic lines (97.5 ± 2.0% for *CprISPS-60* and 99.4 ± 1.2% for *CprISPS-68*) relative to Col-0 plants (94.4 ± 5.5%). By contrast, in the case of the 300 mM mannitol treatment, a very significant increase of seed germination was observed in both transgenic lines (98.1 ± 3.8% and 98.1 ± 1.3% for both *CprISPS-60* and *CprISPS-68*) compared with Col-0 wild-type plants (88.8 ± 3.2%; *p* = 0.009457 and 0.006137, respectively, based on Student’s *t*-test) (Figure 3A,B). Consistent with seed germination rates, the cotyledon greening rates of all lines growing in the normal conditions were similar, while the cotyledon greening rates of *CprISPS-60* (97.5 ± 2.0%) and *CprISPS-68* (98.8 ± 1.4%) were only slightly higher than that of Col-0 wild-type plants (92.5 ± 4.6%, *p* > 0.05) when treated with 200 mM mannitol. Under the treatment with 300 mM mannitol, the cotyledon greening rates of *CprISPS-60* (71.9 ± 4.3%) and *CprISPS-68* (86.9 ± 8.0%) were significantly higher than that of WT (50.0 ± 4.3%; *p* = 0.001852 and 0.000713, respectively, with Student’s *t*-test) (Figure 3C).

### 2.3. Overexpression of CprISPS Enhances Drought Stress Tolerance at Post-Germination Stage

**Figure 4:**
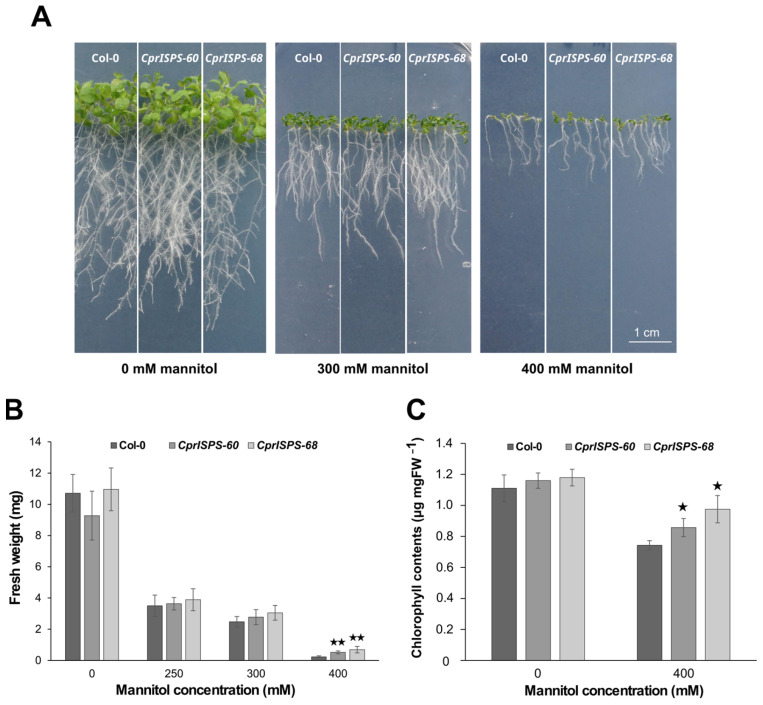
Effect of mannitol treatment on seedling growth of transgenic lines overexpressing the *CprISPS* gene. (**A**) Phenotype variations among Col-0 and two transgenic lines growing in different concentrations of mannitol. (**B**) Fresh weight of Col-0 and transgenic plants growing on different concentrations of mannitol (0 mM, 250 mM, 300 mM, 400 mM) for 10 days. (**C**) Chlorophyll contents of Col-0 and transgenic plants growing on different concentrations of mannitol (0 mM, 400 mM) for 10 days. Histogram bars shown with stars are significantly different from Col-0 (★ statistically significant at *p* < 0.05; ★★ statistically significant at *p* < 0.01). Error bars represent the SD of the means. Scale bar = 1 cm.

To further explore the drought responses induced by mannitol at the post-germination stage, the phenotypic variations of *CprISPS* transgenic lines and Col-0 wild-type plants were observed after vertically growing them on medium supplemented with 0 mM, 250 mM, 300 mM, or 400 mM mannitol for 10 days. The plant growth was assessed in terms of inhibition by mannitol of root elongation and shoot growth (Figure 4A). Root elongation did not show any obvious differences between the *CprISPS* and WT lines under any of the concentrations of mannitol tested here. Instead, shoot growth was significantly higher for both *CprISPS* transgenic lines compared with that of Col-0 wild-type plants treated with 400 mM mannitol, which was also reflected by their fresh weight. In the 400 mM mannitol treatment, the average fresh weight of the WT line was 0.23 ± 0.06 g/plant, while the weights of *CprISPS-60* and *CprISPS-68* were 0.51 ± 0.09 g/plant and 0.69 ± 0.21 g/plant, respectively, more than twice of that of Col-0 (*p* = 0.00051 and *p* = 0.00144, respectively, based on Student’s *t*-test; Figure 4B).

As an effective indicator of drought stress responses [54], the chlorophyll content of *CprISPS* transgenic lines was compared to that of Col-0 wild-type plants. Under control condition (0 mM mannitol), the chlorophyll contents of *CprISPS-60* (1.160 ± 0.05 µg/mg), *CprISPS-68* (1.180 ± 0.05 µg/mg), and Col-0 wild-type plants (1.111 ± 0.09 µg/mg) did not differ significantly from each other. However, when treated with 400 mM mannitol for 10 days, the chlorophyll contents of CprISPS-60 (0.857 ± 0.06 µg/mg) and CprISPS-68 (0.976 ± 0.09 µg/mg) were significantly higher than that of Col-0 wild-type plants (0.743 ± 0.03 µg/mg, Student’s *t*-test *p*-value < 0.05 for both comparisons) (Figure 4C).

### 2.4. Overexpression of CprISPS Induces the Expression of Stress-Related Genes

**Figure 5:**
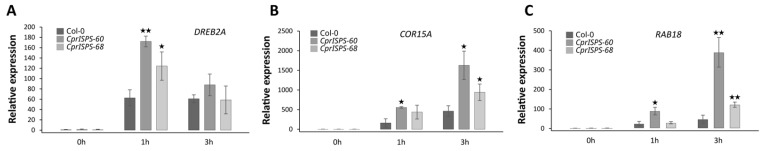
Expression pattern of drought-responsive genes under mannitol stress treatment. (**A**–**C**) qRT–PCR analysis of drought-responsive genes in *CprISPS* transgenic plants compared with Col-0 under mannitol stress at different time-points. Histogram bars marked with stars are significantly different from Col-0 (★ statistically significant at *p* < 0.05; ★★ statistically significant at *p* < 0.01). Error bars represent the SD of the means.

To further understand the drought resistance of *CprISPS* transgenic lines at the molecular level, the expression levels of key stress-related marker genes (*AtDREB2A*, *AtCOR15A* and *AtRAB18*) were studied by qRT-PCR, using *AtEF1α* and *AtGAPDH* as reference genes (Figure 5). After mannitol treatment for a specific time (1 h, 3 h), the aerial parts were collected. Under control conditions (0 mM mannitol), the expression of *AtDREB2A*, *AtCOR15A,* and *AtRAB18* were similar among *CprISPS-60*, *CprISPS-68* transgenic and WT plants. Under mannitol treatment (500 mM), the expression of *AtDREB2A* in the aerial part of the overexpressor plants increased significantly compared with that of Col-0 for 1 h, but there were no differences among different genotypes after 3 h mannitol exposure (Figure 5A). For the expression patterns of *AtCOR15A* and *AtRAB18*, similar expression trends were observed (Figure 5B,C). The expression level of only one transgenic line (*CprISPS-60*) was significantly increased after 1 h treatment, while it significantly increased for *AtCOR15A* and very significantly for *AtRAB18* for both transgenic lines compared with that of Col-0 after 3 h treatment.

### 2.5. Overexpression of CprISPS Increases Drought Tolerance at the Adult Stage

**Figure 6:**
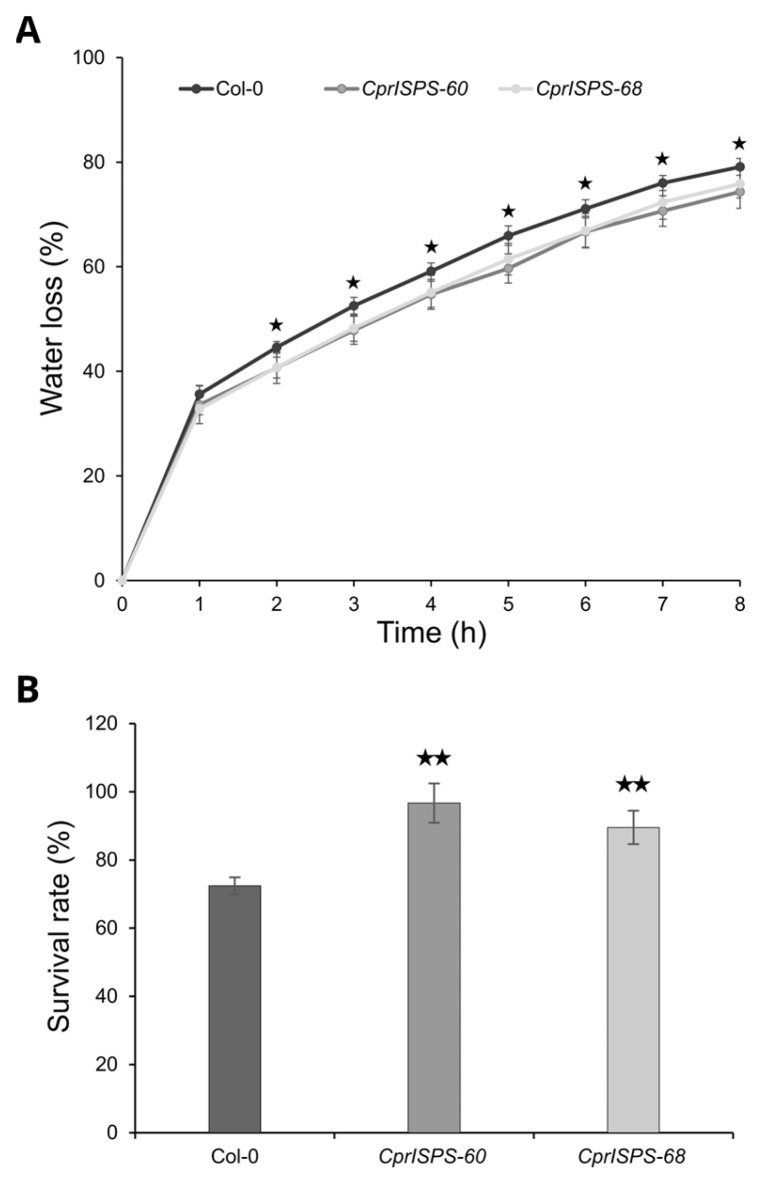
Water loss and survival analyses of Arabidopsis plants overexpressing *CprISPS*. (**A**) Water loss from the leaves of Col-0 and transgenic plants at various time-points after leaf detachment. Asterisks represent significant differences between Col-0 and the transgenic lines (*t*-test, *p* < 0.05). Line bars show the standard deviation of the mean. (**B**) Survival rate among Col-0 and two transgenic lines was recorded after four-day recovery by withholding water when the soil water content in the pots was around 20–25%. Histogram bars marked with the star show significant difference from Col-0 (★ statistically significant at *p* < 0.05; ★★ statistically significant at *p* < 0.01). Line bars represent the SD.

To investigate the drought responses at the adult developmental stage, the water loss rate from detached leaves and the survival rate in soil by withholding water were determined using three-week-old plants. After being detached from plants, the water loss rates of mature rosette leaves of both transgenic lines (33.6 ± 3.6% for *CprISPS-60* and 32.9 ± 1.2% for *CprISPS-68*) were not distinguishable from that of Col-0 wild-type plants (35.6 ± 1.7%, Student’s *t*-test *p* > 0.05) at the first hour. From the second hour on, however, the water loss rates of transgenic lines were significantly lower than that of WT plants (Student’s *t*-test *p* < 0.05 for all data points). At the 8th hour, the water loss rates of *CprISPS-60* and *CprISPS-68* were 74.3 ± 3.2% and 75.8 ± 2.7%, respectively, while a water loss of 79.1 ± 1.6% was observed for Col-0 wild-type plants (Figure 6A). Also, the survival rate of whole plants exposed to prolonged water stress in soil were enhanced in *CprISPS* overexpressing lines compared to WT. After re-watering for 4 days, the survival rates of *CprISPS-60* and *CprISPS-68* were 96.7 ± 5.8% and 89.5 ± 4.9%, respectively, significantly higher than that of WT (72.4 ± 2.5%; *p* = 0.00262, 0.00649 Student’s *t*-test) (Figure 6B). Taken together, the water loss rate and survival rate analyses suggested that the *ISPS* gene from *C. prunifera* enhanced drought stress responses in transgenic Arabidopsis not only during the early developmental stages, but also at the adult developmental stage.

## 3. Discussion

The fact that isoprene emission and drought stress reciprocally influence each other in plants is well established. However, while the consequences of drought stress on the biosynthesis and emission of isoprene have been elucidated in detail, e.g., [38,55,56,57,58,59,60], the physiological and, even more, the molecular mechanisms that underlie the capacity of isoprene to mitigate the negative effects that water deprivation has on plant growth and development [38,40,61,62] are still only partially understood. As ISPS is the only enzyme that is both necessary and sufficient to engineer isoprene emission in non-emitting species, heterologous expression of *ISPS* genes from natural isoprene emitters in *A. thaliana* is a powerful and widely adopted approach that can provide deeper functional insights on the relevance of isoprene emission in the donor species by validating ISPS function and comparing the effects of presence/absence of the trait in a uniform genetic background (e.g., [16,41,43]). In this study, given the high species richness of Arecaceae in neotropical dry forests [63] and more generally in seasonally dry habitats [64], we tested the hypothesis of whether part of the *C. prunifera* capacity to withstand extended periods of drought stress could be due to the isoprene produced by the *CprISPS* isolated in our laboratory. As *C*. *prunifera* lacks established protocols of genetic transformation, we cloned and overexpressed *CprISPS* in *A. thaliana* for the following: (1) to validate it is a functional isoprene synthase; (2) to investigate whether it can enhance drought tolerance; (3) whether tolerance persists throughout plant growth and development or is stage-specific; (4) to determine what are the transcriptional responses and the possible involvement of selected key genes mediating such capacity.

### 3.1. Common and Special Features of CprISPS

Contrary to some *ISPS* genes from other Arecaceae species preliminary characterized in our laboratory, the *CprISPS* characterized in this study provided a high level of isoprene emission and degree of tolerance to drought when overexpressed in Arabidopsis. This interspecific variation is in line with the variable levels in isoprene emission capacity observed in vivo in palms [48,65]. Indeed, even congeneric species of the most commonly studied isoprene-emitting dicotyledonous trees such as oaks and poplars as well as monocotyledonous *Arundo* species (e.g., [2,12,66] displayed variable emission levels of isoprene. With the data currently available, it is unfortunately not possible to pinpoint the possible cause(s) for the observed differences among Arecaceae ISPS to induce enhanced drought tolerance when overexpressed in Arabidopsis. In fact, CprISPS appears to be a typical ISPS (Figure 1) for the following reasons: (1) it has the two conserved Phe residues F312 and F459 (homologous to F338 and F485 in *Populus alba*) which are the marker residues for all confirmed isoprene synthase in angiosperms [16,67]; (2) it shares with all other ISPS from the same family the same Arecaceae-specific diagnostic tetrad of residues (FVFT), the conserved aspartate-rich, metal-binding motif D319DXXD of terpenoid cyclases as well as the other metal-binding motif N463DXXXXXXE [48]. Hypothesizing that a minimal isoprene dosage threshold necessary to trigger enhanced drought tolerance may exist in Arabidopsis, a possible reason for the variable capacity of Arecaceae ISPS to enhance drought tolerance could be interspecific differences in ISPS catalytic activities. However, a multitude of biological, environmental or simply technical factors are known to affect the measurements of isoprene emission from single plant species, making it virtually impossible to establish whether differences in enzymatic activity are a major cause underlying the significant emission differences reported in vivo at genus and family levels [68]. Therefore, in the future it will be important to systematically test the threshold hypothesis, for instance, by exploiting the large numbers of transgenic lines with nearly continuous levels of isoprene emission that are generated in a uniform genetic background using the same binary transformation vector in this and previous studies [16,46,48,69].

### 3.2. Isoprene-Induced Increase of Drought Tolerance Persists throughout Plant Growth and Development

With the notable exception of gray poplar transgenic lines with *ISPS* downregulation [70], the capacity of isoprene to enhance drought tolerance in studies of both natural and especially engineered isoprene emitters from both monocots and dicots (e.g., [37,38,40,45,71,72] suggests that this trait may be independent of the particular plant species considered. As the majority of these previous studies mainly focused on investigating the physiological responses at a specific stage of plant growth, in this study we focused on the isoprene-related physiological and molecular changes related to drought tolerance at different developmental stages because drought stress is known to inhibit seed germination, seedling root elongation, shoot growth, and biomass production [73,74]. The results we obtained here clearly point to isoprene-mediated enhanced drought resistance throughout the whole plant life cycle of *CprISPS* transgenic Arabidopsis. In particular, we observed alleviation of drought stress of *CprISPS* transgenic plants at the germination and early post-germination stages in terms of increased seed germination (Figure 3A,B) and cotyledon greening rate (Figure 3C). The lack of effects on germination in ABA-treated Arabidopsis overexpressors of *AdoISPS* [46] suggest that *CprISPS* overexpression enhancement of germination in the presence of mannitol may be not mediated by ABA at this stage. On the other hand, we cannot currently exclude that cotyledon-enhanced greening in the overexpressors may be at least in part mediated by ABA or simply by a more advanced developmental stage caused by earlier germination. However, the most parsimonious hypothesis for this phenotype is possibly the capacity of isoprene to increase chlorophyll content that has been repeatedly and consistently reported across different species [43,75,76,77]. Recent proteomic and metabolomic studies indicate that the higher chlorophyll content of isoprene emitting plants, however, is just the tip of an iceberg of isoprene-mediated mechanisms of drought tolerance, which collectively could explain all the phenotypes observed at the later developmental stages in *CprISPS* overexpressor plants. In particular, the higher chlorophyll content (Figure 4) is consistent with the ability of drought-stressed Arabidopsis isoprene emitters to avoid the negative effects of this type of stress on photosynthesis through a direct or, more likely, indirect antioxidant action of isoprene [41,78]. On the other hand, *CprISPS* enhanced biomass accumulation (Figure 4b) is consistent with previous observations that only isoprene emitting lines transformed with *Eucalyptus globulus ISPS* can maintain leaf tissue growth comparable to non-drought stressed plants [41]. As this phenotype was significant in the experimental conditions used in our study only under the most severe stress applied, future work will be necessary to test whether isoprene emission can improve plant growth in more subtle ways also at lower levels of drought stress. The lower water loss and higher survival upon prolonged drought stress observed in this study are fully consistent with the previous report that mature isoprene emitting plants showed enhanced ABA-induced stomatal closure [46], suggesting that in this case ABA likely contributes to enhance water stress tolerance. In addition to an increased ABA content in leaves of *ISPS* overexpressors [41], the increased abundance of genes for the biosynthesis of osmoprotectants and redox homeostasis compounds like trehalose and proline causes a significant increase, especially, of the former compound in drought stressed *Arabidopis* engineered to emit isoprene [41].

### 3.3. Induction of Expression Levels of Key Drought Regulators Are Mediated by Isoprene

Analysis of the expression levels of key drought stress-related genes in the aerial part of water-stressed plants provides further insight on the mechanism underlying the above physiological observations. In the aerial part, in fact, the expression of *RAB18* (At5g66400), *COR15A* (At2g42540), and *DREB2A* (At5g05410) were up-regulated in *CprISPS* transgenic lines compared to Col-0 under mannitol treatment. Among these genes, *COR15A* is an ABA-dependent dehydration and cold/osmotic-responsive gene [79,80]. The mature *COR15A* protein participates in the protection of chloroplast membranes in freezing-induced dehydration [81], which is consistent with the increase in chlorophyll content of *CprISPS* transgenic plants and confirms its upregulation in isoprene emitting Arabidopsis plants under drought stress [46]. Additionally, *COR15A* is coordinately upregulated together with the two dehydrin genes *RD29B* and *RAB18* in Arabidopsis transgenic lines overexpressing a *DREB* transcription factor from rice that is able to increase drought, salt, and low temperature stress tolerance in both species under the control of the core ABA signaling pathway [82]. Notably, overexpression of *AdoISPS* in *A. thaliana* has been reported to promote ABA-induced stomatal closure by enhancing the expression of *RD29B* (and to a minor extent *RAB18*), thereby reducing water loss rate under short-term water stress [46]. As *RAB18* can be induced by drought [83] and is highly expressed in guard cells [84], we suggest that the significantly lower water loss rate of *CprISPS* detached leaves may be at least partly mediated by isoprene through the ABA-dependent control of stomatal responses mediated by dehydrins.

As mentioned above, however, we found that also *DREB2A* transcription is upregulated by the combination of isoprene emission and drought stress. *DREB2A* is a key transcription factor in the drought response pathway and can strongly induce the expression of the several LEA/dehydrin genes, among which are *RD29A* and *RD29B,* in an ABA-independent way [85,86]. Although *RD29A* was not upregulated in isoprene-emitting Arabidopsis upon dehydration stress [46], these results indicate that isoprene might be involved in regulating the osmotic stress responses through both ABA-dependent and ABA-independent induction of a subset of dehydrins. As several dehydrins are known to interact with each other as well as with the plasma membrane aquaporin AtPIP2B [87,88], in-depth studies on these two classes of proteins are promising research areas to elucidate the fine details of isoprene-mediated drought tolerance. Worthy of note, in previous transcriptomics studies none of the genes validated here and in [46] were identified to be differentially expressed in response to isoprene in the absence of drought stress [43,89]. This is most likely due to the rapid and transient transcriptional upregulation of the transcription factors involved in early drought stress responses [86] and corroborates the proposed action of isoprene as a signaling molecule, possibly also involved in priming to stress [42,43,44,89].

In summary, the present study confirms and extends previous work on the capacity of isoprene to enhance drought tolerance in the model system *A. thaliana*. In addition to defining novel aspects of the physiological responses involved in the development of drought tolerance at different stages of plant growth and development, these results uncover the capacity of isoprene to mediate the fast and dynamic transcriptional upregulation of dehydrin genes certainly involving ABA-dependent and possibly also ABA-independent pathways. Most importantly, these results suggest that the wide conservation of isoprene emission at high levels throughout the Arecaceae family could have an adaptive role contributing to enhance drought tolerance to levels which offset the high metabolic and carbon costs for isoprene biosynthesis. While future studies will be required to fully elucidate the gene regulatory cascades responsible for this isoprene-mediated trait, the *ISPS* gene from *C. prunifera* characterized in this study shows already promise for the engineering of more drought-tolerant and climate-change resilient crops.

## 4. Materials and Methods

### 4.1. Plant Materials and Growth Conditions

*Copernicia prunifera* (Mill.) H. Moore, *Arabidopsis thaliana* L. (from now on for simplicity referred to as Arabidopsis) Col-0 wild-type plants and two T4 homozygous transgenic lines (*CprISPS-60*, *CprISPS-68*), overexpressing the isoprene synthase from *C. prunifera* driven by CaMV 35S promoter, were used in this study. The expression of the *CprISPS* transgene was confirmed by semi-quantitative RT-PCR using *AtEF1α* as control (Supplementary Figure S1). The primers used are listed in Supplementary Table S1. *C. prunifera* plants were grown in the Botanic Garden of Padova University (Italy). Arabidopsis seeds were sown on Murashige–Skoog (MS) agar medium containing 1% sucrose after surface-sterilization with 70% ethanol and 5% household bleach according to [47]. After stratification at 4 °C for three days, the seedlings were cultivated in a growth chamber at 23 °C with a relative humidity of 60% and light intensity of 100–120 µmol m^−2^ s^−1^ under a 16 h light/8 h dark photoperiod.

### 4.2. Genomic DNA Purification, Total RNA Isolation and cDNA Synthesis

Genomic DNA purification was based on the simplified CTAB method using around 100 mg of fresh leaf material. The extracted genomic DNA was checked in 0.8% Agarose gel and analyzed using Quant-iT™ dsDNA Assay Kit (ThermoFisher, Waltham, MA, USA) for quantification.

The Trizol reagent (Invitrogen™, Waltham, MA, USA) was applied for total RNA extraction with around 100 mg of frozen plant material, and the trace of genomic DNA contamination was eliminated with Amplification-Grade DNaseI (Sigma-Aldrich^®^, St. Louis, MO, USA). The integrity and quality analyses of isolated total RNA and cDNA synthesis using SuperScript™III Reverse Transcriptase (Invitrogen™) were performed according to the former description by [90].

### 4.3. Cloning, Plasmid Constructs, Plant Transformation, and Screening

For the isolation of the novel isoprene synthase gene from *C. prunifera*, the same cloning approach previously described [48] was applied to identify the 5′ and 3′ untranslated regions of the gene using genomic DNA extracted from *C. prunifera* leaves as a template. Thus, the full-length cDNA of *CprISPS* was amplified using the synthesized cDNA as mentioned above from *C. prunifera* leaves with Q5^®^ High-Fidelity DNA Polymerase (New England BioLabs, Ipswich, MA, USA) and a pair of primers designed according to the sequences derived from the genome walker method. The primers used for these amplifications are listed in the Supplementary Table S1. The *CprISPS* sequence was deposited under accession number OQ557194 in GenBank.

The amplified full-length *CprISPS* cDNA was cloned into the pENTR/D-TOPO vector and recombined into the destination vector pK7WG2 through LR reaction with LR clonase II (Invitrogen^TM^) under the control of constitutive cauliflower mosaic virus (CaMV) 35 S promoter [91]. This construct was first transformed into *A. tumefaciens* strain GV3101-pMP90RK by electroporation and afterwards into the *A. thaliana* Col-0 wild-type plants by the floral dip method [92]. T1 transgenic plants were screened on 1/2 MS solid medium supplemented with 50 mg/L kanamycin. The homozygous single-copy T4 seeds from two selected lines were used for all downstream experiments. All sequences used here were obtained by sequencing with a 96-capillary 3730xl DNA Analyzer (Thermo Scientific, Waltham, MA, USA).

### 4.4. PTR-MS Measurement

Isoprene emissions of transgenic plants were screened with proton transfer reaction–mass spectrometry (PTR-MS) as previously described [69]. Briefly, a rosette leaf was detached with a pair of scissors from one-month-old plants, weighed, and transferred into a 20 mL glass vial containing 300 µL of distilled water. Before capping, the vials were kept open for 30 min and then incubated at 30 °C for 3 h under the light intensity of 130–150 µmol m^−2^ s^−1^. Isoprene emission measurement was conducted with PTR-ToF 8000 apparatus (Ionicon Analytik GmbH, Innsbruck, Austria).

### 4.5. Seed Germination and Green Cotyledon Assays

The surface-sterilized seeds were grown on 1/6 MS medium supplemented with 200 and 300 mM mannitol for seven days for seed germination rate and cotyledon greening rate assays. Seed germination was recorded as in [46], when the radicle penetrated the seed coat completely, and the cotyledon greening was recorded when the cotyledon was open and green. Four replicates were conducted, and 40 seeds were used for each replicate.

### 4.6. Young Seedling Fresh Weight Measurement

Col-0 and *CprISPS* transgenic seeds were surface sterilized according to [47] and sown on 1/6 MS medium containing 0, 250 mM, 300 mM, or 400 mM mannitol and grown vertically for 14 days. The phenotypes were photographed, and the fresh weight of young seedlings was measured. At least 50 plants were used for this assay and four replicates were performed.

### 4.7. Chlorophyll Content Analysis

Seven-day-old Col-0 and *CprISPS* transgenic seedlings growing on 1/6 MS medium were transferred to fresh 1/6 MS medium containing 0 mM or 400 mM mannitol and cultivated vertically for an additional 10 days. The aerial part was collected and used for chlorophyll content analyses. Chlorophyll contents were determined as previously reported [43,46]. Briefly, the samples were homogenized using a tissue lyser with 800 µL of 80% ice-cold acetone. After centrifugation at 4600 rpm at 4 °C for 13 min, the supernatant was transferred to a fresh tube. The pellet was resuspended with another 400 µL of 80% ice-cold acetone and centrifuged as before. The absorbance of the combined supernatants was measured at 663 nm and 646 nm using an Ultrospec 3100 proUV/Visible Spectrophotometer (GE Healthcare, Chicago, IL, USA). According to Lichtenthaler and Wellburn [93], the total chlorophyll content was calculated as: C (µg/mL) = 7.18 A_663_ + 17.32 A_646_. The final chlorophyll concentration of each sample was normalized according to the fresh weight. Four replicates were performed for this analysis.

### 4.8. qRT-PCR Analysis

Ten-day-old Col-0 wild-type and *CprISPS* transgenic seedlings growing on 1/6 MS medium were transferred onto fresh 1/6 MS medium containing 0 mM or 500 mM mannitol. Afterwards, the aerial parts and roots were separately harvested at different time points (0 h, 1 h, and 3 h), snap-frozen in liquid nitrogen and stored at −80 °C. The qRT-PCR was performed with Platinum SYBR Green qPCR SuperMix-UDG (Invitrogen). The reaction system and program were performed according to the previous method [94]. At least three technical and three biological replicates were conducted for each sample. *AtEF1α* and *AtGAPDH* were selected as internal reference genes for mannitol treatment, and *AtEF1α* used for semi-quantitative analyses of Arabidopsis transgenic lines. The relative transcription levels of these genes were analyzed by the 2^−ΔΔCt^ method. The primers used for qRT-PCR are listed in Supplementary Table S1.

### 4.9. Water Loss Rate Evaluation

Seven-day-old Col-0 and *CprISPS* transgenic seedlings growing on 1/2 MS medium were transferred into commercial soil and grown in the same conditions as mentioned above. After two-week growth, the mature rosette leaves were detached from plants and the fresh weight was measured and recorded immediately, and then the detached leaves were kept at room temperature for eight hours and the fresh weight measurement was carried out every one hour. The water loss rate was calculated as the percentage of the fresh weight difference between each indicated time versus the initial fresh weight. For each genotype, five replicates were performed. Each replicate included 16 rosette leaves of similar size detached from 8 plants.

### 4.10. Survival Rate Assay

For the survival rate determination under drought treatment, the two *CprISPS* transgenic lines and Col-0 wild-type plants were grown under normal conditions as mentioned above for three weeks. Then, the plants were deprived of water by withholding water when the soil humidity of each pot reached 20–25%, followed by re-watering for four days. Three replicates were performed for this analysis and each replicate contained 20 plants. The soil humidity was calculated as follows: soil humidity = (wet soil mass − dry soil mass)/wet soil mass × 100%.

### 4.11. Statistical Analyses

For statistical analyses of differences of experimental results, the Student’s *t*-test was carried out for all the assays. A threshold of *p* < 0.05 was applied to determine statistical significance and *p* < 0.01 for highly significant difference.

## Data Availability

All the data related to this work are included in the article or in the Supplementary Materials.

## Supplementary Materials

The following supporting information can be downloaded at: https://www.mdpi.com/article/10.3390/ijms242015329/s1.
