# Supplementary material for: A Novel Isoprene Synthase from the Monocot Tree Copernicia prunifera (Arecaceae) Confers Enhanced Drought Tolerance in Transgenic Arabidopsis
Source: Int J Mol Sci. 2023 Oct 18;24(20):15329. doi: 10.3390/ijms242015329 (PMC10607627; doi:10.3390/ijms242015329)

A novel isoprene synthase from the monocot tree *Copernicia prunifera* (Arecaceae) confers enhanced drought tolerance in transgenic Arabidopsis.

Yu Jiamei[1,2], Iuliia Khomenko[3], Franco Biasioli[3], Mingai Li[1,4] and Claudio Varotto[1,4]

[1] Biodiversity, Ecology and Environment Area, Research and Innovation Centre, Fondazione Edmund Mach, via Mach 1, 38098, San Michele all'Adige, Trento, Italy

[2] Department of Biology, University of Padova, Via Ugo Bassi 58B, Padova 35121, Italy

[3] Food and Nutrition Area, Research and Innovation Centre, Fondazione Edmund Mach, via Mach 1, 38098, San Michele all'Adige, Trento, Italy

[4] NBFC, National Biodiversity Future Center, Palermo 90133, Italy

Supplementary Table S1: Primers used in this study.

Primer name	Sequence (5' - 3')	Purpose
EguISPS_GW-Rev1	GGAAACCCGTGCTCTCTAAGGAGTC	GenomeWalker
EguISPS_GW-Rev2	GTCATCTTTGAACATCAAGTCTGCCCTC	GenomeWalker
ISPS_For	CCAAACAATAGAAGTAGCTCAAGC	full-length cDNA cloning
ISPS_Rev	CCCTTCTACACATTGCTGACC	full-length cDNA cloning
CprISPS_For	CACCATGGCATTCTCTACATGCTTCG	pENTR cloning
CprISPS_Rev	CTACACATTGCTGACCACCAACCT	pENTR cloning
pK7WG2_For	GACAATCTGATCCAAGCTCAAGC	Screening for transgene
CprISPS_RT_For	CAGCAATCAATCTTGGACGG	Semi-quantitative RT-PCR
CprISPS_RT_Rev	CTACACATTGCTGACCACCA	Semi-quantitative RT-PCR
DREB2A_RT_For	CTACAAAGCCTCAACTACGGAATAC	qRT-PCR
DREB2A_RT_Rev	AAACTCGGATAGAGAATCAACAGTC	qRT-PCR
COR15A_RT_For	GATACATTGGGTAAAGAAGCTGAGA	qRT-PCR
COR15A_RT_Rev	ACATGAAGAGAGAGGATATGGATCA	qRT-PCR
RAB18_RT_For	GGAAGAAGGGAATAACACAAAAGAT	qRT-PCR
RAB18_RT_Rev	GCGTTACAAACCCTCATTATTTTTA	qRT-PCR
AtEF1α_RT_For	TGAGCACGCTCTTCTTGCTTTCA	qRT-PCR; Semi-quantitative RT-PCR
AtEF1α_RT_Rev	GGTGGTGGCATCCATCTTGTTACA	qRT-PCR; Semi-quantitative RT-PCR
AtGAPDH_RT_For	TTGGTGACAACAGGTCAAGCA	qRT-PCR
AtGAPDH_RT_Rev	AAACTTGTCGCTCAATGCAAT	qRT-PCR

Supplementary Figure S1: Expression levels (semi-quantitative RT-PCR) of the *CprISPS* transgene in the two Arabidopsis transgenic lines used in this study for all experiments. The gene *EF1α* from Arabidopsis (*AtEF1α*) was used as normalization control.

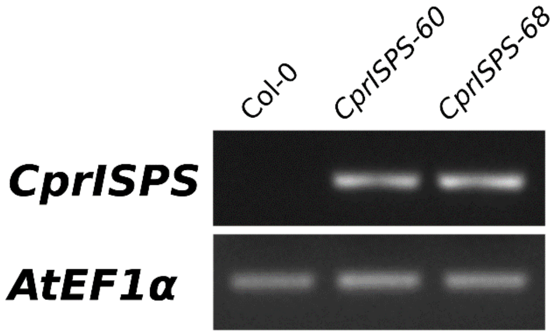

Supplement: Supplementary file 1 [file ijms-24-15329-s001.zip › ijms-2609691-supplementary.pdf]
